# Assessment of Women Physicians Among Authors of Perspective-Type Articles Published in High-Impact Pediatric Journals

**DOI:** 10.1001/jamanetworkopen.2018.0802

**Published:** 2018-07-20

**Authors:** Julie K. Silver, Julie A. Poorman, Julia M. Reilly, Nancy D. Spector, Richard Goldstein, Ross D. Zafonte

**Affiliations:** 1 Department of Physical Medicine and Rehabilitation, Harvard Medical School, Boston, Massachusetts; 2 Department of Pediatrics, Drexel University College of Medicine, Philadelphia, Pennsylvania

## Abstract

**Importance:**

Most pediatricians are women; however, women pediatricians are underrepresented in academic leadership positions such as department chairs and journal editors and among first authors of original research articles published in pediatric journals. Publication of all types of articles, particularly in high-impact specialty journals, is crucial to career building and academic success.

**Objective:**

To examine the gender-related profile associated with authors of perspective-type articles in the 4 highest-impact general pediatric journals to determine whether women physicians were similarly underrepresented.

**Design and Setting:**

Cross-sectional study of perspective-type articles published between 2013 and 2017 in the 4 highest-impact general pediatric journals: *Academic Pediatrics*, *JAMA Pediatrics*, *The Journal of Pediatrics*, and *Pediatrics*.

**Main Outcomes and Measures:**

The primary outcome measure was the number and percentage of first-author women physicians as compared with men physicians. Secondary outcome measures included number and percentage of all men and all women among last authors and coauthors associated with physician first authors.

**Results:**

A total of 425 perspective-type articles were identified, with physicians listed as the first author on 338 (79.5%). Women were underrepresented among physician first authors of known gender (140 of 336 [41.7%]), particularly among physician first authors of article categories described as scholarly (range, 15.4%-44.1%) vs categories described as narrative (range, 52.9%-65.6%) in nature. Women were also underrepresented among last authors and coauthors of articles attributed to both men and women physician first authors, although the underrepresentation of women among last authors and coauthors was more pronounced if a man physician was the first author.

**Conclusions and Relevance:**

Because perspective-type articles provide an opportunity for authors to express their opinions, provide insights that may influence their field, and enhance their academic resumes, there is a need for pediatric journal editors and leaders of medical societies who are associated with these journals to ensure the equitable inclusion of women in medicine. A hallmark of best practices for diversity and inclusion in academic medicine is transparency with regard to reporting of gender disparities in all areas of scholarship attribution and credit.

## Key Points

**Question:**

Are women, who in 2015 made up 61.9% of pediatricians and 53.0% of full-time physician pediatric faculty, underrepresented among physician first authors of perspective-type articles published in the 4 highest-impact general pediatric journals?

**Findings:**

In this cross-sectional study, women were underrepresented among physician first authors (140 of 336 [41.7%]). Underrepresentation was more pronounced in article categories described as more scholarly (range, 15.4%-44.1%) vs narrative (range, 52.9%-65.6%).

**Meaning:**

Because women are underrepresented among physician first authors of perspective-type articles, they are less likely to have opportunities to express their opinions and provide insights that may influence the field.

## Teaser

This study assesses the proportion of women physician authors of perspective-type articles in 4 high-impact pediatric journals in comparison with the proportion of men physician authors of these articles.

## Introduction

Pediatrics is a specialty in which women outnumber men. In 2015, the proportion of women among active pediatricians in the United States was 61.9%,^1^ yet women held just 53.0% of full-time physician pediatric faculty positions (with 32% at the rank of full professor)^2^ and 20% of chair positions.^3^ Additionally, a recent study by Fishman et al^4^ found that women continue to be underrepresented among authors of original research publications and editors associated with 3 high-impact pediatric-focused journals (*Pediatrics, JAMA Pediatrics,* and *The Journal of Pediatrics*).

In this study, we examined whether the underrepresentation of women in pediatrics extended to perspective-type articles in peer-reviewed journals, as these types of articles, in contrast to editorials, commentaries about articles in the journal, and original research studies, do not necessarily require expertise in a specific subfield of study. Perspective-type articles may be written from a more general point of view by physicians at any stage of their professional career. However, this unique category of articles provides opportunities for physicians to express their opinions, providing insights that may both influence the field and enhance their professional reputations. To our knowledge, this study is the first of its kind in the medical literature.

## Methods

This is a cross-sectional study of authorship of perspective-type articles in high-impact pediatric journals published during a 5-year period (January 1, 2013, to December 31, 2017). Journals were included if they were among the top 25 pediatric journals as ranked by *InCites Journal Citation Reports* 2016 impact factor, they focused on general pediatrics, and their online list and description of article categories included an independent opinion and/or perspective-type article. Article categories described as written by the editorial staff were excluded. Categories described as editorials or commentaries related to specific published reports were also excluded, as critique at this level generally narrows the pool of potential authors to physicians with a focused area of research and/or other academic work. The journals (and categories) included were *Academic Pediatrics* (Perspective and In the Moment), *JAMA Pediatrics* (Viewpoint and On My Mind), *The Journal of Pediatrics* (Commentary), and *Pediatrics* (Pediatrics Perspective).

### Outcomes

The main outcome measures were numbers and proportions of men and women among physician first authors. Secondary outcome measures included numbers and proportions of men and women among last authors and coauthors of articles written by physician first authors. Authors counted as physicians included authors with credentials identifying them as such (eg, MD, DO, and MBBCh). Gender was determined by inspection of authors’ first and middle names followed by an internet search for information, including photographs that depicted the author as a man or woman and/or profiles that used terminology such as *male, female, man, woman, he,* or *she.* The genders of 4 authors (2 first authors and 2 last authors) could not be determined. Articles for which first-author gender could not be determined were excluded. Articles for which last-author gender could not be determined were excluded from analyses related to coauthors and last authors. This report conforms to the Strengthening the Reporting of Observational Studies in Epidemiology (STROBE) reporting guideline for reporting of cross-sectional studies. Because the information contained within was publicly available, the Partners Healthcare internal review board determined that review was not required.

### Statistical Analysis

Statistical analysis of underrepresentation consisted of an exact binomial test comparing the proportion of women among physician first authors of perspective-type articles with the proportion of women in active pediatric practice in 2013 (60.4%)^5^ and 2015 (61.9%)^1^ and women among physician full-time pediatric faculty in 2014 (52.0%)^6^ and 2015 (53.0%)^2^ as reported by the Association of American Medical Colleges (AAMC). The test was performed using the Stata statistical software version 15.1 (StataCorp) command *bitest* and treating the population proportion as a given, or gold standard. The confidence intervals for the proportions of women among physician first authors were calculated using Stata command *ci*. The confidence intervals for the differences were calculated following the formula for Wald confidence intervals.^7^

## Results

**Figure 1.**
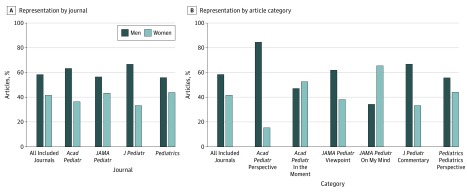
Representation of Women Among Physician First Authors of Perspective-Type Articles The graphs shows percentages of articles written by men vs women physicians by journal (A) and article category (B).

A total of 425 articles were identified. Of these, physicians were listed as the first author on 338 (79.5%). After exclusion of 2 physician first authors of unknown gender, men and women were found to be physician first author of 196 (58.3%) and 140 (41.7%) of the 336 articles, respectively (Figure 1A). When compared with recent proportions of women in active pediatric practice in 2013 (60.4%)^5^ and 2015 (61.9%)^1^ and women among physician full-time pediatric faculty in 2014 (52.0%)^6^ and 2015 (53.0%),^2^ women were found to be underrepresented among physician first authors, with proportions in the 4 individual journals ranging from 33.3% (14 of 42) in *The Journal of Pediatrics* to 44.1% (41 of 93) in *Pediatrics*. Two categories of articles were included from *Academic Pediatrics* and *JAMA Pediatrics,* and subsequent analysis examined each article category separately. The percentage of women among physician first authors of articles published in *Academic Pediatrics* decreased from 36.7% (11 of 30) with combined article categories (Figure 1A) to 15.4% (2 of 13) for the Perspective category alone (Figure 1B). Similarly, the percentage of women among physician first authors of articles published in *JAMA Pediatrics* decreased from 43.3% (74 of 171) with combined article categories (Figure 1A) to 38.1% (53 of 139) for the Viewpoint category alone (Figure 1B). In contrast, the percentage of women among physician first authors in *Academic Pediatrics* increased from 36.7% (11 of 30) with combined categories (Figure 1A) to 52.9% (9 of 17) for the In the Moment category alone (Figure 1B). In *JAMA Pediatrics*, the percentage of women among physician first authors increased from 43.3% (74 of 171) with combined categories (Figure 1A) to 65.6% (21 of 32) for the On My Mind category alone.

Examination of the article category descriptions published in each journal’s instructions for authors^8,9,10,11^ revealed that *Academic Pediatrics’* Perspective and *JAMA Pediatrics’* Viewpoint categories were described similarly to *The Journal of Pediatrics’* Commentary and *Pediatrics’* Pediatrics Perspective categories, as more scholarly and focused on important topics in pediatric medicine (Table 1). In all cases, women among physician first authors of perspective-type articles described as more scholarly were underrepresented (range, 15.4%-44.1%). In contrast, *Academic Pediatrics’* In the Moment and *JAMA Pediatrics’* On My Mind categories were described as more narrative and personal in nature. Women among physician first authors of these narrative perspective-type articles were more equitably represented (range, 52.9%-65.6%). Although public profiles were incomplete, article categories with the lowest representation of women among physician first authors were also those associated with defined editorial staff-driven solicitation processes (*Academic Pediatrics’* Perspective at 15.4% [2 of 13] and *The Journal of Pediatrics’* Commentary at 33.3% [14 of 42]), journals with the lowest 2013 to 2016 *InCites Journal Citation Reports* impact factors (*Academic Pediatrics’* at 2.007-2.720 and *The Journal of Pediatrics’* at 3.736-3.890), and men listed as category editors (*Academic Pediatrics’* Perspective). Finally, all 4 journals listed men as editors in chief.^12,13,14,15^

**Table 1.** Representation of Women Among Physician First Authors and the Article Category’s Information

Journal Title and Article Category	Women Among Physician First Authors, %	Brief Category Description	Solicitation Process	Editors	*InCites Journal Citation Reports* Impact Factor
*Academic Pediatrics* Perspective^8^	15.4	“Perspectives presents important pediatric topics… and identifying areas for future study.” “Authors will generally be respected authorities in the area and may include a fellow or junior faculty member as a co-author.”	“The Perspectives Editors solicit most articles with input about topics and potential authors from the Journal's senior editorial group.”	Chief: man Perspective: 2 men In the Moment: 1 woman	2017: NA 2016: 2.720 2015: 2.438 2014: 2.007 2013: 2.227
*Academic Pediatrics* In the Moment^8^	52.9	“In the Moment is a forum for authors to relate their personal experience of pediatrics. We are seeking narrative pieces about research, contact with patients, the influence of mentors, the impact of policy and current events, and the relationship of the author's work to their lives and the lives of others.”	“We invite submissions to In the Moment…”		
*JAMA Pediatrics* Viewpoint^9^	38.1	“These papers may address virtually any important topic in medicine, public health, research, discovery, prevention, ethics, health policy, or health law and generally are not linked to a specific article. Viewpoints should be well focused, scholarly, and clearly presented.”	Not specifically described	Chief: man Viewpoint: NL On My Mind: NL	2017: NA 2016: 10.251 2015: 9.528 2014: 7.148 2013: NA
*JAMA Pediatrics* On My Mind^9^	65.6	“Most essays published in On My Mind are personal vignettes…taken from wide-ranging experiences in medicine; occasional pieces express views and opinions on the myriad issues that affect the profession.”	Not specifically described		
*The Journal of Pediatrics* Commentary^10^	33.3	“Commentaries should serve as a forum for governmental health policies, economic issues, medical/scientific ethics, psychosocial issues, and international health, particularly in the developed world.”	“Authors who wish to propose a Commentary must e-mail a proposal letter and formal academic outline of the manuscript (i.e., introduction, thesis statement, supporting ideas, and conclusion), identifying the article type for the Editors to assess…”	Chief: man Commentary: NL	2017: NA 2016: 3.874 2015: 3.890 2014: 3.790 2013: 3.736
*Pediatrics* Pediatrics Perspective^11,12^	44.1	“Pediatrics Perspectives…focus on issues of policy, public health, or other research and clinical topics”	“Pediatrics Perspectives are unsolicited opinion pieces…”	Chief: man Pediatrics Perspective: NL	2017: NA 2016: 5.705 2015: 5.196 2014: 5.473 2013: 5.297

Abbreviations: NA, not available; NL, not listed.

**Figure 2.**
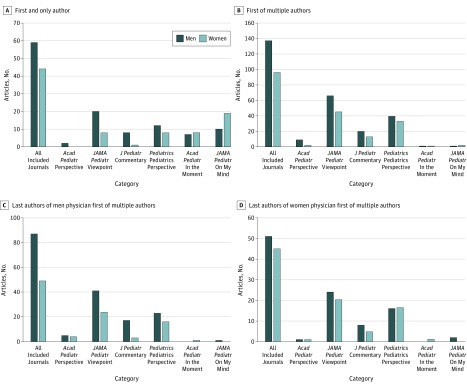
Gender-Related Representation Associated With Physician First Authors of Perspective-Type Articles, 2013-2017 The graphs show the proportion of men vs women who were physician first and only author (A), physician first of multiple authors (B), last author of articles with a man as physician first of multiple authors (C), and last author of articles with a woman as physician first of multiple authors (D).

Physician first authors were further subdivided into 2 groups: (1) first and only authors and (2) first of multiple authors (Figure 2A and B). Women were underrepresented among physician first and only authors overall (42.7% [44 of 103]) as well as among physician first and only authors of the scholarly perspective-type articles (*Academic Pediatrics’* Perspective, 0% [0 of 2]; *JAMA Pediatrics’* Viewpoint, 28.6% [8 of 28]; *The Journal of Pediatrics’* Commentary, 11.1% [1 of 9]; and *Pediatrics’* Pediatrics Perspective, 40.0% [8 of 20]). Women were also underrepresented among physician first of multiple authors overall (41.2% [96 of 233]) and among physician first of multiple authors of the scholarly perspective-type articles (*Academic Pediatrics’* Perspective, 18.2% [2 of 11]; *JAMA Pediatrics’* Viewpoint, 40.5% [45 of 111]; *The Journal of Pediatrics’* Commentary, 39.4% [13 of 33]; and *Pediatrics’* Pediatrics Perspective, 45.2% [33 of 73]). In contrast, women were equitably represented or overrepresented among physician first and only authors of the more narrative *Academic Pediatrics’* In the Moment articles (53.3% [8 of 15]) and *JAMA Pediatrics’* On My Mind articles (65.5% [19 of 29]) as well as among physician first of multiple authors of the narrative perspective-type articles (range, 50%-66.7%), although these constituted a small number of articles (n = 2-3).

To examine the gender-related representation of last authors, physician first of multiple authors were further subdivided by gender (Figure 2C and D). Women were underrepresented among last authors of articles written by men physician first authors overall (36.0% [49 of 136]). Moreover, women were underrepresented among last authors of articles written by men physician first authors in the 4 individual scholarly perspective-type article categories (*Academic Pediatrics’* Perspective, 44.4% [4 of 9]; *JAMA Pediatrics’* Viewpoint, 37.9% [25 of 66]; *The Journal of Pediatrics’* Commentary, 15.0% [3 of 20]; and *Pediatrics’* Pediatrics Perspective, 41.0% [16 of 39]). Likewise, although less pronounced, women were underrepresented among last authors of articles written by women physician first authors overall (46.9% [45 of 96]). Separately, women were underrepresented among last authors of articles written by women physician first authors in the scholarly *JAMA Pediatrics’* Viewpoint (46.7% [21 of 45]) and *The Journal of Pediatrics’* Commentary (38.5% [5 of 13]). Notably, among authors of the scholarly perspective-type articles published in *Pediatrics’* Pediatrics Perspective, women physician first of multiple authors were more equitably associated with women last authors. Analysis of gender representation among last authors of *Academic Pediatrics’* scholarly Perspective articles written by women as well as the narrative perspective-type articles, although shown in Figure 2C and D, were inconclusive because of small sample sizes (*Academic Pediatrics*’ Perspective articles written by women physician first authors [n = 2], all *Academic Pediatrics*’ In the Moment articles [n = 2], and all *JAMA Pediatrics’* On My Mind articles [n = 3]).

The significance of the underrepresentation of women among physician first authors was determined by comparing physician first authorship by article category within individual journal-years (Table 2) with the respective representation of women among physicians in active pediatric practice^1,5^ and among full-time physician pediatric faculty^2,6^ as reported by the AAMC. In only 2 years did the percentage of women among physician first authors of the scholarly perspective-type articles meet or surpass the threshold of their reported representation within the specialty and among full-time physician pediatric faculty: 2013 and 2015 *Pediatrics’* Pediatrics Perspective. In contrast, the percentage of women among physician first authors of narrative perspective-type articles surpassed the threshold of their reported representation within the specialty and among full-time physician pediatric faculty 5 times: in 2014 and 2017 for *Academic Pediatrics’* In the Moment and in 2015, 2016, and 2017 for *JAMA Pediatrics’* On My Mind. Notably, although data from the AAMC indicated increased percentages of women both in active pediatric practice and full-time physician faculty, only in *JAMA Pediatrics’* On My Mind did the percentage of women among physician first authors increase each year during the study period. Moreover, in 3 of the 5 years studied, *Academic Pediatrics* published no scholarly Perspective category articles written by women physician first authors.

**Table 2.** Gender-Related Representation Among Physician First Authors of Perspective-Type Articles by Journal and Year, 2013-2017

Year	Woman Physician First Authors, No. (% [95% CI])^a^	AAMC Physicians in Active Pediatric Practice			AAMC Physicians Among Full-Time Pediatric Faculty		
		Women, No. (%)^b^	Difference (95% CI)	*P* Value^c^	Women, No. (%)^b^	Difference (95% CI)	*P* Value^d^
**All Included Journals and Article Categories**							
2013	16 of 47 (34.0 [20.9-49.3])	33 944 (60.4)	26.4 (12.8-40.0)	<.001	NA		
2014	23 of 61 (37.7 [25.6-51.0])	NA			7938 (52)	14.3 (2.1-26.5)	.03
2015	34 of 71 (47.9 [35.9-60.1; 35.9-60.1])	35 573 (61.9)	14.0 (2.4-25.6)	.02	9250 (53)	5.1 (−6.6 to 16.8)	.41
2016	29 of 78 (37.2)	NA			NA		
2017	38 of 79 (48.1)	NA			NA		
***Academic Pediatrics’* Perspective**							
2013	1 of 3 (33.3 [0.8-90.6])	33 944 (60.4)	27.1 (−26.1 to 80.3)	.57	NA		
2014	0 of 3 (0 [0-70.8])	NA			7938 (52)	52.0 (50.9-53.1)	.11
2015	0 of 2 (0 [0-84.2; 0-84.2])	35 573 (61.9)	61.9 (41.0-82.8)	.15	9250 (53)	53.0 (52.0-54.0)	.22
2016	1 of 3 (33.3)	NA			NA		
2017	0 of 2	NA			NA		
***JAMA Pediatrics’* Viewpoint**							
2013	7 of 20 (35.0 [15.4-59.2])	33 944 (60.4)	25.4 (4.5-46.3)	.02	NA		
2014	9 of 22 (40.9 [20.7-63.6])	NA			7938 (52)	11.1 (−9.5 to 31.7)	.39
2015	13 of 30 (43.3 [25.5-62.6; 25.5-62.6])	35 573 (61.9)	18.6 (−3.1 to 40.3)	.04	9250 (53)	9.7 (−8.1 to 27.5)	.36
2016	12 of 34 (35.3)	NA			NA		
2017	12 of 33 (36.4)	NA			NA		
***Journal of Pediatrics’* Commentary**							
2013	3 of 10 (30.0 [6.7-65.2])	33 944 (60.4)	30.4 (2.0-58.8)	.06	NA		
2014	1 of 4 (25.0 [0.6-80.6])	NA			7938 (52)	27.0 (−15.4 to 69.4)	.36
2015	3 of 9 (33.3 [7.5-70.1; 7.5-70.1])	35 573 (61.9)	28.6 (−2.2 to 59.4)	.09	9250 (53)	19.7 (−11.1 to 50.5)	.32
2016	6 of 14 (42.9)	NA			NA		
2017	1 of 5 (20.0)	NA			NA		
***Pediatrics’* Pediatrics Perspective**							
2013	4 of 7 (57.1 [18.4-90.1])	33 944 (60.4)	3.3 (−33.4 to 40.0)	>.99	NA		
2014	7 of 21 (33.3 [14.6-57.0])^e^	NA			7938 (52)	18.7 (−1.5 to 38.9)	.12
2015	15 of 24 (62.5 [40.6-81.2; 40.6-81.2])	35 573 (61.9)	−0.6 (−20.0 to 18.8)	>.99	9250 (53)	−9.5 (−28.9 to 9.9)	.42
2016	4 of 17 (23.5)	NA			NA		
2017	11 of 24 (45.8)^e^	NA			NA		
***Academic Pediatrics’* In the Moment**							
2013	1 of 3 (33.3 [0.8-90.6])	33 944 (60.4)	27.1 (−26.2 to 80.4)	.57	NA		
2014	3 of 4 (75.0 [19.4-99.4])	NA			7938 (52)	−23.0 (−65.4 to 19.4)	.63
2015	1 of 3 (33.3 [0.8-90.6; 0-70.8])	35 573 (61.9)	28.6 (−24.7 to 81.9)	.56	9250 (53)	19.7 (−76.3 to 30.3)	.60
2016	0 of 3	NA			NA		
2017	4 of 4 (100)	NA			NA		
***JAMA Pediatrics’* On My Mind**							
2013	0 of 4 (0 [0-60.2])	33 944 (60.4)	60.4 (59.9-60.9)	.02	NA		
2014	3 of 7 (42.9 [9.9-81.6])	NA			7938 (52)	9.1 (−27.6 to 45.8)	.72
2015	2 of 3 (66.7 [9.4-99.2; 9.4-99.2])	35 573 (61.9)	−4.8 (−58.1 to 48.5)	>.99	9250 (53)	−13.7 (−67.0 to 39.6)	>.99
2016	6 of 7 (85.7)	NA			NA		
2017	10 of 11 (90.9)	NA			NA		

Abbreviations: AAMC, Association of American Medical Colleges; NA, not applicable.

^a^ When 2 sets of confidence intervals are listed, the confidence intervals correspond to the comparisons of the percentage of women among physician first authors with (1) the percentage of women among physicians in active pediatric practice and (2) the percentage of women among physician full-time pediatric faculty as reported by the AAMC, respectively.

^b^ Data are not available for some years because AAMC does not report these data every year.

^c^ Represents the significance of the underrepresentation of women when comparing the percentage of women among physician first authors of perspective-type articles and the percentage of women in active pediatric practice as reported by the AAMC.

^d^ Represents the significance of the underrepresentation of women when comparing the percentage of women among physician first authors of perspective-type articles and the percentage of women among physician full-time pediatric faculty as reported by the AAMC.

^e^ One physician of unknown gender was excluded from analysis.

**Figure 3.**
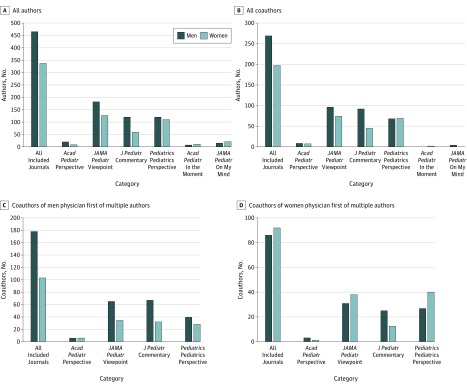
Gender-Related Representation Among Coauthors Associated With Physician First Authors of Perspective-Type Articles, 2013-2017 The graphs show proportion of men vs women for all authors of articles with a physician as first author (A), all coauthors of articles with a physician as first author (B), coauthors of articles with a man as physician first author (C), and coauthors of articles with a woman as physician first author (D).

Gender-related representation was also examined among all authors and coauthors of perspective-type articles. Overall, women accounted for 337 of 802 authors (42.0% [range, 31.0%-58.3%]) associated with physician first author articles included in this study (Figure 3A). The highest proportions of women among all authors were seen in the narrative perspective-type article categories, with *Academic Pediatrics’* In the Moment (57.9% [11 of 19]) and *JAMA Pediatrics’* On My Mind (58.3% [21 of 36]). Similarly, women accounted for 197 of 466 coauthors (42.3% [range, 0%-100%]) associated with physician first author articles included in this study (Figure 3B). Subsequent analysis of coauthor gender revealed that women were underrepresented among coauthors of scholarly perspective-type articles written by men physician first authors (36.7% [103 of 281] [range, 32.3%-50%]) (Figure 3C). In contrast, women were more equitably represented among coauthors of scholarly perspective-type articles written by women physician first authors overall (51.7% [92 of 178]) and within *JAMA Pediatrics’* Viewpoint (55.1% [38 of 69]) and *Pediatrics’* Pediatrics Perspective (59.7% [40 of 67]) categories (Figure 3D).

## Discussion

In the 4 highest-impact general pediatric journals containing perspective-type articles, covering a specialty in which, as of 2015, the majority of physicians (61.9%)^1^ and faculty (53.0%)^2^ are women, our analysis revealed that women were underrepresented among (1) physician first authors overall; (2) last authors of articles attributed to both men and women physician first authors, although the underrepresentation of women among last authors was more pronounced for men physician first authors; and (3) coauthors of articles attributed to men physician first authors. Notably, the gaps were less pronounced among (1) physician first authors of perspective-type articles described as more personal and narrative in nature and (2) coauthors of articles attributed to women physician first authors in *JAMA Pediatrics’* Viewpoint and *Pediatrics’* Pediatrics Perspective categories. Two journals had both narrative and scholarly perspective-type articles and in both journals, the gaps in representation were more pronounced for the scholarly articles. Prior studies have documented similar gender-related gaps in journal publications,^16,17,18,19^ including in pediatric journals.^4,20^ However, to our knowledge, this is the first study focused on the gender associated with physician authors of perspective-type articles. This study contributes to the existing literature by highlighting that the breadth of the gender-related publication gap is not limited to articles focused on original research.

Although causality was not investigated, the underrepresentation of women physicians cannot reasonably be attributed to a lack of qualified candidates because during the study period, 2013 to 2017, the number of women physicians in active pediatric practice was more than 33 000^5^ and more than 7000^21^ of them were full-time academic faculty. We suggest that one possible conclusion is that there may be a flawed process at the journal level related to institutional bias. Institutional bias is defined as a “tendency for the procedures and practices of particular institutions to operate in ways which result in certain social groups being advantaged or favoured and others being disadvantaged or devalued. This need not be the result of any conscious prejudice or discrimination but rather secondary to the majority following existing rules or norms.”^22^

Institutional bias is consistent with a growing body of literature that refutes lack of a sufficient pipeline (number of qualified women physicians) or a leaky pipeline (loss of qualified women physicians) as reasonable explanations for gender disparities in the physician workforce. In fact, more than a decade ago Carnes et al^23^ published a report that provided a compelling case as to why pipeline issues were inadequate to explain the underrepresentation of women physicians in academic leadership. More recently, in his 2017 presidential address to the American Surgical Association, Keith Lillemoe, MD, PhD, announced to members that although only 1 woman physician had been president of the organization in its 137-year history, “The number of outstanding, qualified female candidates is more than adequate to fill every open surgical leadership position in America today. The problem is not the pipeline—it is the process.”^24^ Our results similarly suggest that the pipeline is more than adequate to drive equitable representation among lead authors of perspective-type articles in pediatric medical journals; therefore, other factors must be in play.

Notably, Carnes et al^23^ debunked 2 other traditional explanations for why women continue to be underrepresented in leadership positions: that women are not competing for leadership positions and that they lack the requisite skills required for the position. Given that (1) there is a large pool of women among both active pediatricians and full-time pediatric faculty and (2) women are increasingly moving into full-time faculty positions for which they must presumably compete for publication to prepare for future academic promotion, it seems that there should be an abundance of women who have the professional motivation and requisite skills to be lead author on perspective-type articles.

As organizations are made up of individuals, it is important to consider how people’s unconscious bias may inadvertently contribute to institutional bias. The current literature on implicit bias suggests that everyone, including physicians,^25,26,27,28,29^ has unconscious ways of operating that might affect who we value in any process, including the solicitation of authors for perspective-type articles or selection of authors for publication from among those offering unsolicited submissions. Indeed, implicit bias has been cited as a potential underlying factor associated with gender-related workforce disparities,^23,30,31,32,33,34,35,36,37,38^ and, in this case, institutional bias may in part be due to editors’ unconscious preference for men authors. As implicit bias is unconscious, it is imperative to avoid blaming individuals for their inability to recognize it and instead focus on developing metrics-driven processes that support equitable inclusion. Education about implicit bias may help people recognize it in themselves. There is also a growing body of evidence in medicine and other fields indicating that when gender is not known to the evaluator, and the work rather than the worker is assessed, the inclusion of women increases.^39,40^

The well-documented issue of gender disparity on journal editorial boards, including those in pediatrics,^4,18,41,42,43,44,45,46,47,48^ must also be considered. It is particularly challenging to justify these disparities in a specialty in which there is a pool of some 2600 women candidates for these positions consisting of 1600 associate professors and 1000 full professors alone.^2^ In this study, none of the 4 journals employed a woman in the position of editor in chief, and the only journal that listed editors of perspective-type articles on its masthead was *Academic Pediatrics*. *Academic Pediatrics’* more scholarly Perspective category was assigned to 2 editors who were men, did not include any articles written by women physician first authors in 3 of the 5 years studied, and had the lowest percentage of women among physician first authors (15.4% [2 of 13]). However, this category also included the fewest number of articles during the 5-year study period (n = 13). *Academic Pediatrics’* In the Moment category was assigned to 1 woman editor and included a higher percentage of women among physician first authors (52.9% [9 of 17]). While the data in this study are limited and it is not known what impact gender equity at the editorial level would have with respect to disparities in publication of articles by women,^4,18,41,42,43^ a recent report titled “Publishing While Female” suggested that “asymmetric editorial standards and/or biased referee assignment affect women directly…women write more readably during and spend longer in peer review.”^49^

In a recent report, physicians from 4 different medical specialties highlighted the important role societies may play in closing workforce disparities.^50^ A 6-step process and list of quantitative metrics was proposed to help improve the inclusion of women in these organizations. It was suggested that because journals are often associated with medical societies, including all 4 journals included in this study,^51,52,53,54,55^ journal metrics such as the tracking of inclusion data for editorial positions and all assignments of published articles should be included. Based on the results of this study, additional metrics might include assessment of gender in relation to article solicitations, solicitations accepted and rejected, unsolicited submissions, unsolicited submissions accepted and rejected, and, regardless of solicitation status, biased language and time spent in peer review. Interventions to correct disparities might include implicit bias training for editors and reviewers, increasing the solicitation of scholarly perspective-type articles from women physicians, and blind editorial reviews of perspective-type submissions. Because medical societies rely on membership for sustainability, they may have a powerful (ie, financial) incentive to address gender disparities within their own ranks.

Analysis of author lists revealed that women were underrepresented among last authors and coauthors associated with men and, although less pronounced, women physician first authors of scholarly perspective-type articles. Our results suggest that individual implicit bias may be involved in the writing process as well as the editorial and publication process, and authors, senior researchers, and chairs of departments should examine patterns of association.^56,57,58^ Again, given the large and increasing proportion of women in active pediatric practice and among full-time faculty, it seems reasonable to suggest that deans, chairs, and senior authors could contribute to closing this gender-related gap in authorship by seeking out qualified women contributors and facilitating professional collaboration. Indeed, sponsorship, which in this case could include “enhancing their credibility and recognition”^59^ by involving women in the writing process, has been proposed as an intervention to increase the proportion of women serving in the highest levels of academic medicine.

### Limitations

Selection of journals, article categories, and articles to be included in this study were based on information provided on publisher websites, and we cannot account for errors in any of these areas. In addition, although we cannot exclude the possibility of incidental inclusion, article categories were chosen such that regular contributions from editorial staff (which might skew gender representation) would be excluded. Gender was determined by name inspection followed by online search for photographs and/or profiles that portrayed the author as a man or a woman, and we cannot account for errors in the publication of this information.

The significance of the underrepresentation of women among physician first authors was determined by comparing authorship by article category within individual journal-years with the respective representation of women among physicians in active pediatric practice and among full-time physician pediatric faculty. Both were included because neither group alone was a precise benchmark for authors included in this study. Women among full-time physician pediatric faculty included the more likely contributors, those in academic medicine, but excluded those early in their careers, namely residents and clinical physicians without faculty status as defined by the AAMC. Women among physicians in active pediatric practice included residents and clinical physicians who may not have faculty status, but also included clinicians in private practice who may be less likely to contribute articles. Statistical analysis was also limited to the years for which these data were available from the AAMC.

## Conclusions

Diversity of thought is important for innovation in all fields in medicine. Moreover, the ability of women physicians to voice their opinions and share their knowledge is a critical component of career advancement. This study highlights an opportunity for pediatric journal editors, medical society leaders, and institutional leaders to take steps to ensure the equitable inclusion of women physicians.^50^ This would involve investigating inclusion, determining gaps, implementing strategies to address disparities, tracking results, and reporting outcomes to all stakeholders. While women pediatricians are the primary stakeholders, others include, but are not limited to, chairs of pediatrics departments, deans of medical schools, and leaders of health care institutions who are charged with the equitable advancement and compensation of physicians. Transparency to all stakeholders regarding reporting disparities, interventions, and outcomes is a hallmark of best practices for diversity and inclusion efforts.
